# How to deal with sickness absence among primary school pupils? Adaptation of the “Medical Advice for Sick-reported Students” intervention

**DOI:** 10.3389/fpubh.2023.1139752

**Published:** 2023-11-23

**Authors:** Esther K. Pijl, Yvonne T. M. Vanneste, Jolanda J. P. Mathijssen, Frans J. M. Feron, Angelique E. de Rijk

**Affiliations:** ^1^Child and Youth Healthcare Department, GGD West-Brabant, Breda, Netherlands; ^2^Department of Social Medicine, Faculty of Health Medicine and Life Sciences, Care and Public Health Research Institute (CAPHRI), Maastricht University, Maastricht, Netherlands; ^3^Dutch Knowledge Centre for Youth Health (NCJ), Utrecht, Netherlands; ^4^Academic Collaborative Centre Youth, Tranzo, Tilburg University, Tilburg, Netherlands

**Keywords:** intervention mapping, sickness absence, primary education, primary school pupils, school absenteeism, school attendance, child and youth healthcare, Medical Advice for Sick-reported Students

## Abstract

**Background:**

Missing school impacts both education and health. The purpose of this study was to address sickness absence in primary schools by adjusting the ‘Medical Advice for Sick-reported Students’ intervention for secondary schools. This was necessary because of fundamental differences in relation to the children’s age and in the schools’ organizational structure.

**Methods:**

The intervention mapping approach steps 1 through 4 were used to adapt ‘Medical Advice for Sick-reported Students’ to primary schools (MASS-PS), including a literature search, stakeholder interviews, establishing a planning group and pre-testing.

**Results:**

In step 1, a planning group was formed and a logic model of the problem was created. In step 2, a logic model of change was created. In step 3, a theoretical basis and practical strategies were determined. In step 4, practical support materials were designed, and two pre-tests of the materials were performed.

**Conclusion:**

Intervention mapping was successfully used to adapt MASS to primary schools. The main changes were the lowering of the threshold for extensive sickness absence, consultations between teacher and attendance coordinator, and addition of two experts. With MASS-PS, sickness absence can be addressed as a “red flag” for underlying problems.

## Introduction

Education is crucial for a child’s healthy development. Missing school frequently can lead to lower educational achievement, early school dropout and health problems (1–4). Most types of absenteeism, i.e., truancy, tardiness and sickness absence, have been shown to affect children negatively (5). Research in the Netherlands and England suggests that primary school children miss on average 2–4 out of 100 school days and sickness absence is the most common type of school absenteeism (6–8). This study focuses on adapting an intervention to address sickness absence among primary school pupils.

School attendance is mandatory in the Netherlands from the age of 5 to 16. Sickness absence is defined as absence when a child is reported sick by a parent or legal guardian, for example due to an infectious disease or injury (6, 7). Reporting sick can take place in the absence of physical pathology as well, for example; due to psychological or social problems instead, such as anxiety or bullying. In the Netherlands, sickness absence is considered authorized absenteeism. Further, schools are not obliged to take action for authorized absenteeism (9, 10). In the case of (a suspicion of) unauthorized absenteeism, such as truancy and tardiness, the school has to report this to the school attendance officer, who will investigate the case and can sanction parents on the basis of school attendance being mandatory.

Although it is authorized, frequent and/or longer periods of sickness absence should be addressed by schools. Such extensive sickness absence might be an indication of underlying problems and will have negative consequences for intellectual development and socio-emotional functioning (11, 12). Thus, we start with the assumption that extensive sickness absence requires appropriate action by schools to improve pupils health in its broad sense.

Vanneste et al. (8, 12) have shown that sickness absence can be addressed when they developed an evidence-based approach to sickness absence in secondary education. This ‘Medical Advice for Sick-reported Students’ (MASS) intervention aims to reduce sickness absence through early detection and by providing appropriate care. Students at risk are identified, and then the student, parents and school professionals assess the problem and formulate possible solutions. If the problem is complex or medical, a consultation with a child and youth healthcare physician (CYHP) is scheduled to examine the cause of absenteeism from a biopsychosocial perspective (12, 13). CYHPs are part of the Dutch public health service that supports the healthy development of children. Together, the student, parents, school professionals and CYHP design a plan of action to reduce sickness absence and address underlying causes. Such plans may include optimizing lifestyle, medical treatment and mental health care, or adjusting the school day and changing the lesson plan to fit the individual child’s needs. It may not always be possible to immediately reduce sickness absence, for instance if the absenteeism is necessary for recovery from an illness, but even then a child can be helped, through homework adjustments or online classes for example. The negative consequences of sickness absence may be reduced by ensuring appropriate education and improving the connection to the school and classmates.

This original version of MASS has been successfully adapted to vocational education through the intervention mapping approach (14). Parents were found to have a smaller role than in secondary education, therefore, adaptations were made to improve controlling measures and awareness of the problem among students.

Currently, there is no structural approach to sickness absence in primary schools, even though missing school often starts as early as primary education (6, 15, 16). We decided to adapt the original version of MASS to primary education because early intervention might prevent the development of the problems causing sickness absence. According to the growing-into-deficit concept (8, 17), it is likely that problems that hinder the development of the child at a young age may not yet cluster into a classifiable diagnosis. Early detection of problems, using sickness absence as a ‘red flag’, might provide opportunities to halt the progressive clustering of problems into a disease or disorder, thus improving the child’s development and limiting the need for treatment and health costs in the future (8).

The required adaptation of the original version of MASS was expected to be substantial because of the fundamental differences in the children’s age and in the schools’ organizational structure between primary and secondary education. First, as primary school pupils are younger and less self-sufficient, parents are expected to play a larger role in relation to both the background of sickness absence and reporting sick itself. Second, again because of their age, underlying problems are less likely to have clustered into a classifiable diagnosis. Third, primary schools are often smaller than secondary schools, have fewer teachers per child and are located closer to the child’s home. These differences prompt adapting the original version of MASS for primary schools (MASS-PS) in a systematic way. Similar to the adjustments for vocational education (14), we used the intervention mapping approach to provide a theory- and evidence-based blueprint for intervention development (18). The aim of this study was to adapt MASS to primary education, using intervention mapping.

## Methods

**Figure 1:**
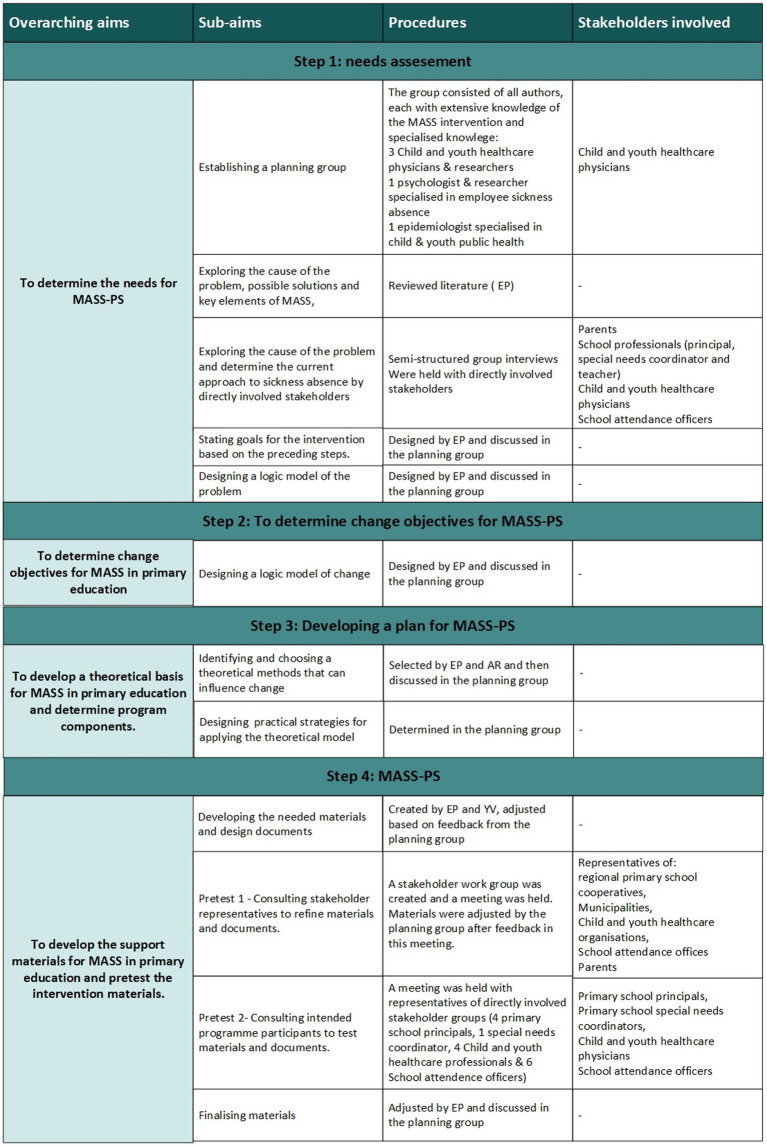
Overview of the aims, goals, procedures and consulted stakeholders during the intervention mapping process used to systematically adapt the Medical Advice for Sick-reported Students intervention to primary schools. MASS-PS: medical advice for sick-reported pupils for primary schools.

Intervention mapping (IM) was used to adapt MASS to primary education in 2017 and consists of six steps to systematically design, implement and evaluate an intervention for health promotion based on empirical, theoretical and practical knowledge (18). In this study, we used steps 1 through 4 to design an intervention, Figure 1 shows an overview of the actions performed. Steps 5 and 6 are reserved for a future study.

### Step 1: needs assessment

The aim of step 1 is to gain insight into the problem of sickness absence in primary education and to determine what is necessary for MASS-PS. This needs assessment was done through establishing a planning group, literature search, interviews with stakeholders, and the development of intervention goals and a logic model of the problem.

The *planning group* was created with researchers in relevant fields to plan and facilitate all the steps of the intervention mapping process and to create the theoretical frame work needed to develop the intervention in step 3 of IM.

For the *literature search w*e used the search engines of Pubmed and EBSCOHost. We searched for literature, including grey literature such as government reports, about school absenteeism or sickness absence in primary education, MASS or other sickness absence interventions in schools, and interventions that address general school absenteeism in primary education. The search terms we used were different combinations of: “Attendance”, “Absenteeism”, “Sickness Absence”, “Sick Leave”, “MASS”, “Medical Advice for Sick-reported Students”, “School”, “Education”, “Primary”, “Elementary”, “Pupil”, and “Student”.

To examine *stakeholders’ views* on causes of sickness absence and necessary improvements for an approach to sickness absence, six semi-structured focus group interviews were held, involving 27 participants from two regions in the Netherlands. The participants represented the stakeholders that are directly involved in addressing sickness absence of primary school children: five parents, five primary school principals, three special needs coordinators and two teachers, as well as seven child and youth healthcare professionals and five school attendance officers. We use the term parents for all primary caregivers of the child, including single parents and guardians. The parents had two or more children in primary education. Most school professionals had over 10 years of experience working in primary schools. The experience of the CHYP varied between less than five years and more than twenty years’ experience. The school attendance officers generally had the least experience of working with primary school pupils, however they, and the CYHPs, had experience working with MASS in secondary and vocational education. The planning group combined the gathered information to determine intervention *goals* and create the *logic model of the problem*. The latter describes the behavioral and environmental determinants of sickness absence. A logic model helps to understand the complexity of a topic and, in this case, is a visual representation of concepts that are relevant causes and consequences sickness absence. A logic model shows the (hypothesized) relation between different concepts (18).

### Step 2: the logic model of change

To determine change objectives for MASS-PS, the planning group developed the *logic model of change* based on the results from step 1. This model describes which behavior or environmental factors need to change to achieve the goal of the intervention.

### Step 3: developing a plan for MASS-PS: theoretical framework and strategies

The planning group chose the theoretical basis and practical strategies to achieve the desired behavioral change described in step 2. When the previous IM steps showed different requirements for primary schools compared to secondary schools, adjustments to the original MASS were made.

### Step 4: MASS-PS

Based on the practical strategies of the previous step, practical support materials were developed by the planning group and were pre-tested during two separate meetings with stakeholders. Pretesting is done in IM to incorporate stakeholders views on the newly developed intervention and materials before finalizing the intervention. This helps to understand if the previous steps have led to an understandable and usable intervention, or if further changes are needed.

#### Primary schools

Primary education in the Netherlands starts at the age of four and generally lasts for eight years. School attendance becomes compulsory from five years of age for all children (9). Dutch primary schools have an average of 210 pupils, although the size can vary depending on the type of education and the school density of an area. Primary education is segregated into regular primary education and special needs education. Schools in the Netherlands are publicly financed, there is no school fee (except for a few private schools). Even so, these ‘public’ Dutch schools are allowed, by law, to have their own foundation regarding religion (e.g., protestant) or educational approach (e.g., Montessori).

For this study, primary school professionals participated in the meetings with stakeholders and to use the MASS-PS intervention after development (to research step 5 and 6 of IM). The school professionals came from 16 regular primary schools that participated in a larger research project exploring sickness absence in primary education in the West-Brabant region of the Netherlands. The 16 schools were located in both urban and rural areas in West-Brabant and, together, had over 3,000 pupils.

## Results

### Step 1: needs assessment

The needs assessment consisted of five aspects: creating a planning group, exploring the literature, interviewing stakeholders, determining goals, and creating a logic model of the problem. Figure 1 shows an overview of the steps and actions performed.

#### Creating a planning group

The planning group was created and consisted of all of the authors, who had diverse expertise on sickness absence and the original MASS intervention.

#### Exploring the literature

Few studies specifically target sickness absence in primary education. Therefore, we also searched the literature on general school absenteeism in primary education and literature on sickness absence in secondary education. We examined both the problems causing absenteeism and the solutions described.

*The literature on the problems associated with general school absenteeism in primary education* revealed the many different factors related to absenteeism, which are often categorized into school environment, home environment and personal factors (2, 19, 20). Focusing on the school environment in primary education, the factors found were: school climate, bullying, school engagement and the connection between teacher and child (2, 19–23). For the home environment, parental involvement, parent’s understanding of the importance of school attendance, mental illness and substance abuse were found to be related to absenteeism, as well as family cohesion, conflict, frequent relocation, language barriers, poverty and low socioeconomic status (2, 19–21, 23). Personal factors, such as a child’s mental problems, can hinder school attendance, while, enjoying school and having a higher academic achievement seemed to boost school attendance (2, 19, 20, 23). A pilot study by Vanneste et al. (8) focused on sickness absence in primary education and found that problems in the home environment were associated with sickness absence of more than nine school days or more than four periods of sickness absence. A period is a separate instance when a pupil is reported sick. A period lasts at least half of the school day. Factors that related to extensive sickness absence in the pilot study were: lack of motivation, incomplete families, families with financial problems, and a mother with a low educational level or without a paid job (8).

*The literature on problems associated with sickness absence in secondary education* revealed causes such as temporary or chronic diseases, injury, and physical and mental health problems. Sickness absence also relates to characteristics of the home environment such as family conflict or a low social economic status, as well as an unhealthy lifestyle, risk behavior, problems at school and an easy attitude towards reporting a child as sick (1, 8, 12, 34).

*The literature on solutions to sickness absence among children* was absent to our knowledge, except for the original MASS intervention (8). This intervention focuses specifically on sickness absence and has a collective and personalized approach (12). Key elements of MASS (25) are:

1. MASS is included in the official school absenteeism protocol.
2. Actions are based on shared responsibility and shared decision-making.
3. The basis for communication is a caring attitude rather than control.
4. School professionals discuss the absenteeism with the parents and student before any further action is planned.
5. A fixed threshold for ‘extensive sickness absence’ is used to target the children at risk, namely more than 7 consecutive days of sickness absence or more than 3 periods in 4 months.
6. The CYHP is informed by the school about the situation before planning a consultation with the student and parents.
7. During the consultation, the CYHP analyses underlying problems from a biopsychosocial perspective, creates an action plan and monitors any planned healthcare steps.
8. School professionals implement and monitor the action plan.

*The literature on solutions to general school absenteeism* revealed factors that have been successful: effective communication between students, parents and teachers; systematic recording and monitoring of absenteeism; assessment of risk and protective factors by professionals; and referring chronically absent students to the right expert (20, 22). Addressing the underlying problems of school absenteeism requires a collaborative effort from the school and social and medical services.

The literature on school absenteeism frequently refers to a three-tiered response to intervention model created by Kearney and Graczyk, which parallels stages of prevention (20, 22, 26–28). Tier 1 efforts are targeted at all students, Tier 2 actions target students at risk, and Tier 3 actions target students who are chronically absent. Key elements are prevention, regular monitoring, early identification of Tier 2 students, and a functional assessment to determine appropriate interventions. The original MASS intervention follows these three tiers with a collective approach (Tier 1) and a more personalized approach when students are more at risk (Tiers 2 and 3). In primary education, Cook et al. (21) developed an intervention targeting truancy based on the three-tiered response model. They found communication between parents and teachers to be crucial for Tier 1. They gave teachers a leading role in Tier 2 and encouraged referral to experts in Tier 3.

#### Stakeholder interviews

Six semi-structured focus group interviews were held with stakeholders who are directly involved with sickness absence among primary school pupils in the Netherlands. The stakeholders consisted of five primary school principals, four special needs coordinators, two teachers, six CYHPs and one nurse, five school attendance officers and five parents of primary school children. Participating professionals worked for or with primary schools in either Amsterdam or West-Brabant region of the Netherlands and the parents had children in primary schools in West-Brabant. A comprehensive description of the methods of the interview study can be found in a separate article, which also includes results (11).

The main message was that all stakeholders believed the child’s wellbeing is very important. The awareness of sickness absence as a threat to the child’s wellbeing was low among school professionals and parents before the interviews. In contrast, school attendance officers and CYHP, who had all worked with MASS, were adamant about the importance of school attendance. School professionals often registered absence. However, they only occasionally used planned steps to address the absence and based the identification of problematic sickness absence on gut-feeling. The stakeholders believed that the causes of sickness absence could be categorized as medical problems, problems at home, problems at school or a combination. Because of the young age of the child, the parents make the decision to report the child as sick. Parents felt helpless about school-related problems. In contrast, school professionals felt capable of addressing school-related problems but regarded problems at home as outside their influence. Additionally, school professionals and school attendance officers often did not see a way to influence medical problems, while CYHPs did. The school professionals preferred to work with experts whom they knew and trusted, without explicitly considering if another professional had more appropriate qualifications.

#### Solutions

Stakeholders felt the need for a clearly structured approach:

1. Registration and monitoring of sickness absence of all pupils.
2. Identifying pupils with problematic sickness absence, either exceeding a threshold or because the teacher noticed problematic absence.
3. Exploring the cause of the absence. In the first instance, the teacher talks to the parents. The participants agreed that a caring, rather than controlling, attitude is crucial. When necessary, the special needs coordinator or principals, who often know both the parents and the child, can support the teacher and parents in these conversations. When the parents and school require further assistance, an additional expert can be included. The two most important experts mentioned were the CYHP and the social worker. The CYHP examines the complex problem of sickness absence from biological, psychological and social angles, advises on re-integration, and can refer the child to psychological or health care. The stakeholders believed social workers could be needed when problems clearly originated in the home environment. In that case, the parents and the social worker were thought capable of starting to address those problems directly, and a broad biopsychosocial analysis was not considered necessary.
4. Addressing underlying problems – tailored to the individual child’s context and in collaboration between the school and parents.
5. Reducing the effects of absenteeism on education, for example through catch-up lessons.
6. Reducing future absence – often through improving the relationship between school professionals and parents.

#### Planning for early intervention

Because of the importance of early intervention expressed both in the literature and among stakeholders, the planning group decided to lower the threshold for extensive sickness absence. The threshold used in MASS for secondary education, of more than seven consecutive days or more than three periods in four months, was considered to be too high to ensure early intervention in primary education. It was deemed more important to include pupils with potential problematic absence, than to keep out pupils without problems, as the tailored intervention was not expected to have negative side-effects. Research that could help determine the best threshold was limited. Based on the pilot study, which showed that pupils with sickness absence of more than 9 days or more than 4 periods in a school year experienced problems, and based on the stakeholders’ idea that more than 3 separate periods of sickness absence may be a sign of underlying problems, we formulated the consensus-based threshold for extensive sickness absence of more than 6 days or more than 3 periods of sickness absence in a school year. Moreover, to allow for early action, the planning group agreed that parents and teachers should be able to trigger action when they expect the sickness absence to be problematic, even if the threshold has not (yet) been met.

#### Goals for the intervention

Based on the needs assessment, we formulated two goals for MASS-PS: firstly, to reduce sickness absence among pupils; secondly, to be able to use sickness absence as a red flag for underlying physical, psychological and/or social problems.

#### Logic model of the problem

**Figure 2:**
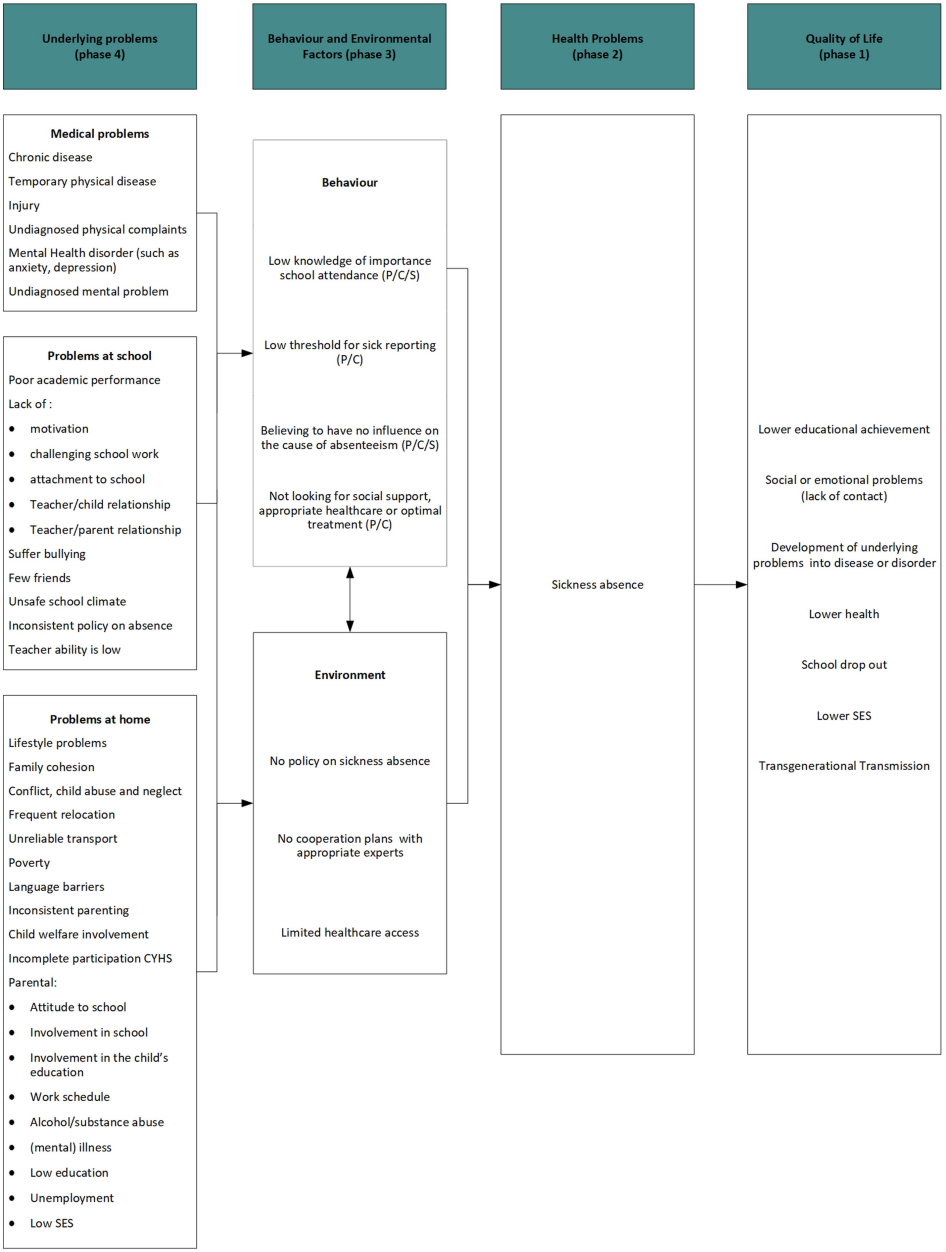
The logic model of the problem for sickness absence in primary education. P: parents, C: child, S: school.

The results of the literature search and stakeholder interviews were combined to create a logic model of the problem that is shown in Figure 2. When creating the model, we started with the concept of quality of life (known as phase 1 in intervention mapping and shown in Figure 2), which may be reduced by sickness absence, which we considered to be the health problem that needs to be addressed (phase 2). Sickness absence in turn is assumed to be influenced by behavioral factors and environmental factors (phase 3). In phase 4 we listed the underlying problems, divided into three categories following the thought process of stakeholders and the literature: medical, school and home problems (Figure 2).

### Step 2: the logic model of change

**Figure 3:**
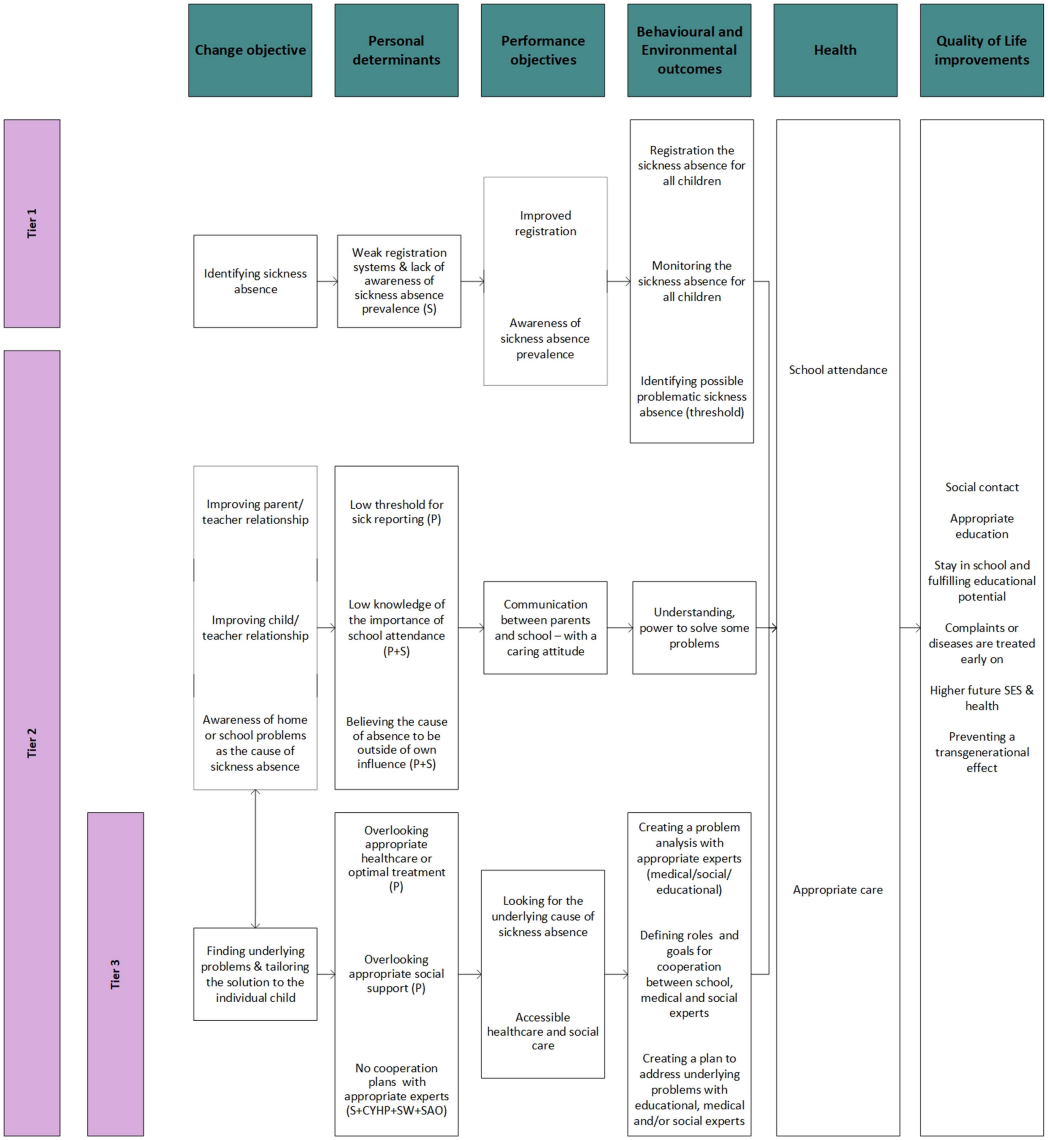
The logic model of change for sickness absence in primary education. CYHP: child and youth healthcare physician, P: parents, S: school, SAO: school attendance officer, SES: Social Economic Status, SW: social worker.

The logic model of change was composed based on the objectives for MASS-PS in addition to the environment and behaviors influencing sickness absence, according to the logic model of the problem which is shown in Figure 3.

### Step 3: developing a plan for MASS-PS: theoretical framework and strategies

The I-change theory was used as a framework to define the behavioral changes of the stakeholders needed to achieve the intervention objectives (29). According to this model, behavior is influenced by ability and motivation. Motivational factors are attitude, social influence and self-efficacy, which can be influenced by awareness. All these determinants are assumed to be important for stakeholder behavior in relation to addressing sickness absence in primary schools.

The theoretical determinants were used to develop strategies and practical applications for each of the key elements of MASS, which can be found in Supplementary material. The focus was put on creating awareness, as the topic of sickness absence as a problem is relatively new to primary schools, and awareness is a crucial step to influence attitude, social influence and self-efficacy.

### Step 4: MASS-PS

#### Developing materials

To visualize MASS-PS we developed a flowchart for professionals to show the stages and their order and who can be involved. Additionally, the threshold for possible problematic sickness absence is shown, as well as advice on communication.

We developed two presentations to share MASS-PS with stakeholders and the participating professionals. The presentations explain why sickness absence needs to be addressed, how MASS-PS was created, and which steps need to be taken to start using MASS-PS.

While developing the materials, our focus was on school professionals because they start the process by registering and identifying problematic sickness absences. They contact the parents and experts and monitor the absence. Therefore, it is important for school professionals to know what to do and why. We created the role of ‘attendance coordinator’ to coordinate the implementation of MASS-PS process in a school. The principal assigned this role to one of the staff members, most often the special needs coordinator. Special needs coordinators are teachers with additional training to examine educational and non-educational needs of individual pupils, which could not be addressed by their own teacher, and to coordinate required actions.

After the initial presentation for all professionals in September of 2017, detailing the IM process from MASS-PS and prototype, the participating schools were visited by a member of the planning group for an hour-long instruction meeting. The main topics addressed during this meeting were prepared beforehand, and there was room for questions. The topics were: the importance of addressing sickness absence, registration, the threshold for extensive sickness absence, the use of a caring attitude, reasons for referrals to experts and contact information for local experts. Each topic was also explained in a leaflet developed for the attendance coordinators. Specific attention was paid to instructing participants to express a caring attitude, to avoid assumptions and judgements, and to finding solutions tailored to individual pupils and their circumstances.

To communicate MASS-PS as a school policy to parents, we created information for the school’s website and newsletter.

Finally, materials from the original MASS were used for the CYHP. All participating child and youth healthcare physicians were trained in MASS consultations by the Netherlands School of Public and Occupational Health in a two-day course to train physicians in examining and supporting sick-reported children.

#### Pretest 1

To pre-test the MASS-PS prototype, we created a group of stakeholder’s representatives. The group was formed through invitations from the regional health office where two planning group members worked at the time. The directors of the three regional school partnerships for primary education in the area participated, as well as the local child and youth healthcare services manager, two representatives of the 18 municipalities in the region, a parent and a CYHP from a different region. The planning group shared the developed MASS-PS materials during a two-hour meeting. The group of representatives emphasized both the importance of registration and its current deficiencies. They believed that the special needs coordinator should be the one to identify and monitor sickness absence, while the teacher is the first one to contact the parents. They agreed that the problem analysis should not be hastily done by the school alone and suggested clarifying the flowchart and adding the role of the school attendance officer to the end. All suggested adjustments were made to MASS-PS.

#### Pretest 2

One month after the first pre-test, a 1.5-h meeting was planned at the regional health office, and end-users were invited. The meeting was attended by four primary school principals, one special needs coordinator, four CYHPs and six school attendance officers. Additionally, the members of the first pre-test group and the planning group also attended this meeting. The adjusted version of MASS-PS was presented and discussed in separate groups. The participants regarded the intervention as logical and feasible, and no new adjustments were made to the flowchart.

#### Finalized materials

**Figure 4:**
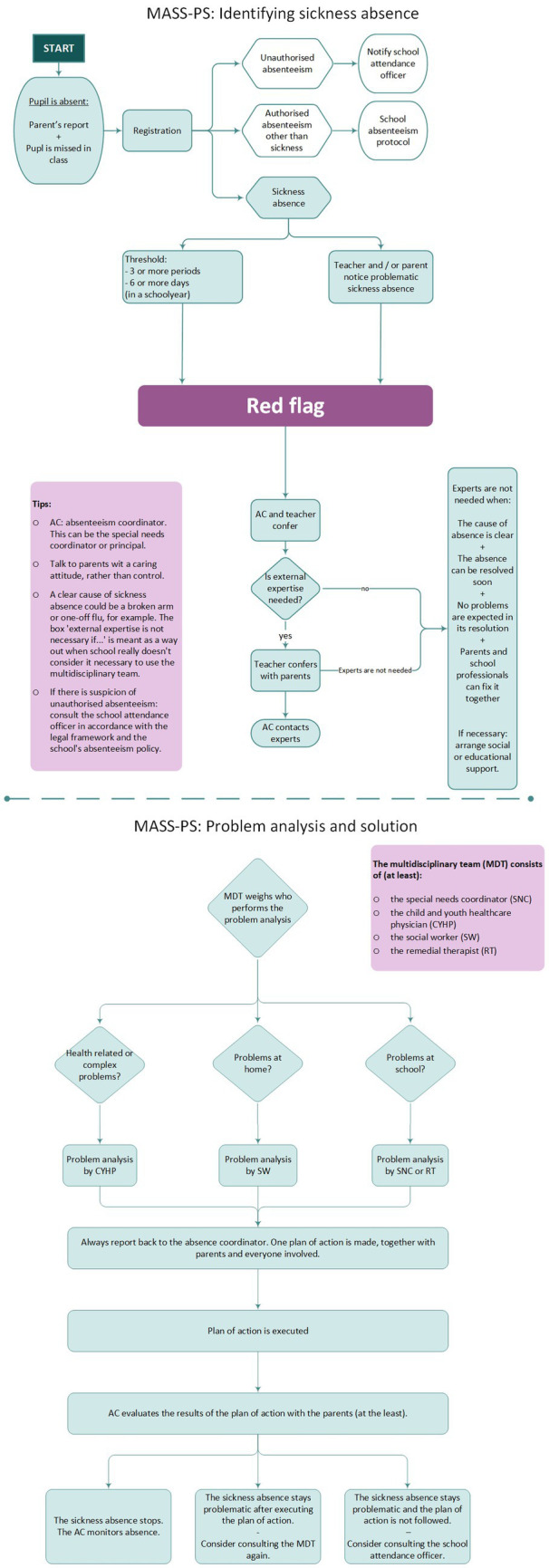
Flowchart of finalized intervention MASS-PS (The Netherlands, 2021).

The finalized flowchart of MASS-PS is shown in Figure 4.

## Discussion

In this study, the original MASS intervention for addressing sickness absence was adapted to primary schools using steps 1 through 4 of the intervention mapping approach (18). The main modifications were the adjustment of the extensive sickness threshold that was used to identify the target group of children, the consultation between the teacher and the attendance coordinator, and the option of referral to the social worker or remedial educationalist as experts in addition to the CYHP.

The threshold used to identify problematic sickness absence in secondary schools was lowered to be able to focus on prevention. Early intervention is necessary to prevent underlying problems from clustering into a disease or disorder and to halt the formation of the habit of missing school. Therefore, sickness absence among primary school children should be seen as a red flag, signaling the need to identify underlying problems and tackle these. Both the sickness absence and the underlying problems impede the development of the child (6, 8).

Teachers, special needs coordinators and principals of primary schools often know the parents. In the Netherlands, parents drop young children off and pick them up at school and also the smaller size of the schools allows for getting to know each other more easily through school activities, as compared to secondary schools where MASS has already been implemented. This implies that all school professionals could have valuable information about the absent child and, therefore, a consultation stage for school professionals was added to MASS. In addition to sharing information about the child, it allows school professionals to share their expertise and confer on their next actions. MASS-PS stresses the parents’ role because the home environment has been shown to have a major impact on attendance (8) and parents are key to the solution. Additionally, due to the child’s age, the parents make most of the decisions regarding appropriate care and reporting their child as sick. Nurturing a good relationship between the parents and the school is thus important to prevent and address sickness absence. Additionally, it has also been shown to improve the child’s academic achievement and mental health (30–32).

Like the original, MASS-PS is offered collectively and provides personalized care for pupils at risk. The actions meant for all children, e.g., a caring approach, registration and identification, could be classified as tier 1 in terms of Kearney and Grazcyk’s intervention model to address school absenteeism (22). The following stages, e.g., problem analysis and solutions, are tailored to the needs of individual children, which may be categorized as tier 2 or 3 interventions. Kearney and Graczyk (33) recently advised the integration of multiple domains of functioning into their three-tiered intervention model, as school absenteeism generally requires a broad perspective. MASS-PS focusses on the school, home and medical domains and incorporates experts in each of these domains, showing that MASS-PS is in line with the current literature on school absenteeism interventions.

### Methodological strengths and limitations

We contemplated several models to adapt MASS to primary schools and their pupils and found that intervention mapping (18) fit best as it led to an in-depth analysis of sickness absence in primary education. Applied Intervention Mapping (AIM), a simplified version of intervention mapping that has been used in the educational setting before (34), was considered, however, we decided a more in-depth analysis was required because of the fundamental differences between primary and secondary schools.

The literature search performed to feed the logic model of the problem was not a systematic literature review and may thus have missed some relevant research. However, as research on sickness absence among primary school pupils is extremely scarce, missing relevant articles seems unlikely. We recommend further research on this topic and on MASS-PS to better understand and address sickness absence. Grey literature was used to incorporate practice-based information.

For the needs assessment we interviewed a large number of directly involved stakeholders, thereby strengthening our practical knowledge. We chose to focus on the stakeholders who have a practical role in addressing sickness absence, rather than those not directly involved, e.g., educational, health care or governmental policymakers. Policymakers were included in the pre-tests to ensure the fit of MASS-PS on a policy level as well as a practical level. The interviewed stakeholders barely touched on the subject of demographic differences between pupils and families when asked about causes of sickness absence among pupils. The interview protocol may not have been specific enough to encourage discussing demographic differences. Literature shows that factors such as poverty, language barriers and low socio-economic status do impact general school absenteeism (1, 2, 19). Further study should thus show in which way demographic differences impact pupils with sickness absence and to what extent these are addressed by the tailored approach of MASS-PS or whether additional elements are required. The I-change theory used has been criticized for its focus on conscious behavior alone (29, 35). There might be additional opportunities to reduce sickness absence if subconscious behavior is targeted. For example, parents and teachers might automatically communicate more easily when they know each other better. This might be promoted by teacher home visits, as has been used previously to address truancy (21).

MASS-PS was tested in two pre-test settings to improve the design. In the second pre-test, no new changes were made to the design, suggesting that MASS-PS is well-suited to the end-users.

The process evaluation of MASS-PS is published elsewhere (36) and the effect evaluation is being prepared.

## Conclusion

Steps 1 through 4 of intervention mapping were successfully used to adapt MASS to primary schools. These IM steps encouraged systematic adaptation of an intervention to address sickness absence in primary schools by combining theory with stakeholders’ input and literature. This new intervention was named MASS-PS.

Compared to the original MASS, adaptations included changes to the threshold for extensive sickness absence, more frequent consultations between teacher and attendance coordinator, and adding the social worker and remedial educationalist as experts along with the child and youth healthcare physician. MASS-PS was well-accepted by professionals in the pre-tests and is ready for the next steps: planning the implementation and evaluation.

The intervention, MASS-PS, can guide school personnel, parents and healthcare professionals and social workers in their combined efforts to address sickness absence among children. By using extensive sickness as a red flag, underlying problems can be addressed, and future health or educational problems can be prevented.

## Data availability statement

The original contributions presented in the study are included in the article/Supplementary material, further inquiries can be directed to the corresponding author.

## Ethics statement

The study was approved of by the Medical Research Ethics Committee (METC) of the Academic Hospital Maastricht/Maastricht University (METC 17-0-114).

## Author contributions

EP, YV, JM, FF, and AR: conceptualization, methodology, funding acquisition, validation, and writing – review & editing. EP, YV, and AR: data curation. EP and AR: formal analysis and investigation. EP: project administration, visualization, and writing – original draft preparation. YV, JM, FF, and AR: supervision. All authors contributed to the article and approved the submitted version.
