# Supplementary material for: How to deal with sickness absence among primary school pupils? Adaptation of the “Medical Advice for Sick-reported Students” intervention
Source: Front Public Health. 2023 Nov 23;11:1139752. doi: 10.3389/fpubh.2023.1139752 (PMC10701280; doi:10.3389/fpubh.2023.1139752)
Supplement: Supplementary file 1 [file Table_1.DOCX]

**Supplementary materials Table 1**. The key elements, strategies and practical applications for the MASS-PS intervention based on theoretical determinants of the I-change theory. (The Netherlands, 2021)

| **Theoretical determinants** | | **Change Objectives** | **MASS version** | **Strategies** | **Practical applications** |
| --- | --- | --- | --- | --- | --- |
| Awareness | Initial information | | Original | Parents should be informed about the use of MASS in their schools, both for transparency and to create awareness of sickness absence as a problem. | Information on school website or newsletter. |
|  | Identification of problematic sickness absence | | Original | Creating awareness about sickness absence through information helps to increase knowledge and risk perception. The importance of addressing sickness absence should therefore be emphasized. | Presentations, topic guides for conversation and written explanation. |
|  |  |  |  | Cues to action should be created, to signal when sickness absence might be problematic. This begins with registration and monitoring of all children’s sickness absences. | Registration and monitoring. |
|  |  |  |  | A threshold can help to identify problematic absence. | Threshold creation for problematic absence |
|  |  |  | New | In primary education, teachers see their pupils frequently and are likely to have a closer relationship with pupils and their parents than teachers in secondary education. The teacher could also notice problematic sickness absence, even before the threshold is reached and should then be able to start the MASS process | Teachers create a cue to action by identifying problematic absence. |
|  |  |  |  | In primary schools, the special needs coordinators and principals know many of the pupils and could have information about them different from that of the teacher. When they share information, awareness of a possible problem will increase. | The absence coordinator consults with the teacher about the sickness absence before a conversation with the parents is planned. |
|  | Examining the underlying cause of sickness absence with experts | | Adjusted | Understanding the added value of additional experts helps the school professionals and parents to find the appropriate care. | Schools are informed about the capabilities of the additional experts and reasons for referral.    Schools inform the parents of the options and refer the parents and child to a consult with the CYHP, social worker and/or remedial specialist.  Additionally, a multidisciplinary meeting can be held to learn about the capabilities of each expert in a specific case. |
| Attitude | Identification of problematic sickness absence | | Original | The attitude of professionals and parents towards addressing sickness absence will be changed through creating awareness by increasing knowledge and risk perception. The importance of addressing sickness absence should be emphasized. | Presentations, topic guides for conversation and written explanation. |
|  | Communication | |  | The attitudes of the school and parents towards addressing sickness absence is also influenced through communication with each other and with experts.  To improve the delivery of the message of sickness absence as a red flag for underlying problems and being a problem itself, the communication should be based on care, rather than control. The aim of communication is shared decision-making with the parents. Communication between the school and parents is crucial for collaboration. | The mindset of communication based on care, rather than control, is included in all MASS-related presentations, conversation guides and written information. |
| Social influence | Identification of problematic sickness absence | | Adjusted | An advocate of addressing sickness absence in the school can encourage registration, identification of problematic sickness absence and the following actions. | Appointment of an absenteeism coordinator at the school.  In primary education, the absence coordinator is the special educational needs coordinator or principal. |
|  |  |  |  | A defined threshold creates a norm and helps to identify problematic sickness absence. The threshold should include lengthy absences and frequent absences, as both could be a sign of underlying problems. The original MASS threshold was adapted for MASS-PS based on the needs assessment and the importance of early intervention to prevent the development of underlying problems. | Define a threshold of six days or three periods of sickness absence in a school year. |
|  | Creating an action plan and evaluating its effects | | Original | The responsibilities regarding sickness absence and its management should be clear. | The parents, child, school and additional experts involved create an action plan and evaluate the plan together. The child and parents follow the action plan by making changes at home and/or following the agreed upon treatments. School professionals implement the educational part of the action plan and monitor sickness absence.  The CYHP monitors the healthcare part of the plan. |
| Self-efficacy | Identification of problematic sickness absence | | New | In primary schools, the special needs coordinators and principals know many of the pupils and could have different information from the teacher. This knowledge about the pupil should be shared. Additionally, the teacher should be facilitated to consider taking measures and asking for advice when needed. | The absence coordinator consults with the teacher about the sickness absence before a conversation with the parents is planned. |
|  | Communication | | Adjusted | School professionals and parents together can take care of some of the problems causing absence. MASS offers an optional communication training for school professionals. This communication training might not be necessary for primary education because the relationship between the parents and teachers is closer, which facilitates good communication.  Additionally, it is common in primary education for the special needs coordinator or principal to support the teacher in conversations with the parents. Special needs coordinators and principals have often been trained in communication. | When there is problematic absence, the teacher and parents sit together to discuss possible solutions.  The special needs coordinator or principal support the teacher in conversations with parents. |
|  | Examining the underlying cause of sickness absence with experts | | Adjusted | The original MASS assumes the CYHP advises the school, parents and child regarding health care and education, to enable the parents, child and school to take appropriate action and reduce sickness absence. The CYHPs are trained to analyze sickness absence from a biopsychosocial perspective together with the child and parents.  For primary education, the needs assessment showed that it was not always preferable to refer all children who meet the threshold to the child and youth healthcare services if the cause of the absence may be a clear problem at home or at school. To tackle such psychosocial problems, the social worker and an education specialist should have a larger role in MASS-PS. | Schools can refer to the CYHP, social worker and/or remedial specialist for further examination of the underlying cause of sickness absence.  After a consult with the CYHP, social worker and/or remedial specialist, the expert informs the absence coordinator of the outcome and, if applicable, refers the child to the appropriate care.  The educational specialist chosen for MASS-PS is a remedial educationalist as they have the appropriate skills and are already affiliated with primary schools. |
|  |  |  | New | Consultation with the additional experts can help the school and parents to determine which additional expert is best suited to perform the problem analysis and to know if the actions taken are right. | MASS-PS includes a multidisciplinary meeting, digitally or physically, initiated by the absenteeism coordinator |
|  | Creating an action plan and evaluating its effects | | Original | When responsibilities of all involved are clear, everyone involved understands their role. | Responsibilities are made clear in MASS |
| Ability | Identification of problematic sickness absence | | Original | Accessible and easy to use registration system is important for consistent registration and identification. | Use of registration software |
|  | Examining the underlying cause of sickness absence with experts | | Original | The CYHP should be trained in using a biopsychosocial approach to sickness absence. | MASS training for CYHP |

MASS: Medical advice for sick-reported pupils

MASS-PS: MASS for primary schools

CYHP: Child and youth healthcare physician
